# Ascochyta Blight in Chickpea: An Update

**DOI:** 10.3390/jof9020203

**Published:** 2023-02-04

**Authors:** Emiliano Foresto, María Evangelina Carezzano, Walter Giordano, Pablo Bogino

**Affiliations:** 1 Instituto de Biotecnología Ambiental y Salud (INBIAS-CONICET), Departamento de Biología Molecular, Facultad de Ciencias Exactas, Físico-Químicas y Naturales, Universidad Nacional de Río Cuarto, Río Cuarto X5804BYA, Córdoba, Argentina; 2 Facultad de Agronomía y Veterinaria, Universidad Nacional de Río Cuarto, Río Cuarto X5804BYA, Córdoba, Argentina

**Keywords:** *Ascochyta rabiei*, *Cicer arietinum*, Ascochyta blight, chickpea defense, disease management

## Abstract

Chickpea (*Cicer arietinum* L.), one of the most cultivated legumes worldwide, is crucial for the economy of several countries and a valuable source of nutrients. Yields may be severely affected by Ascochyta blight, a disease caused by the fungus *Ascochyta rabiei*. Molecular and pathological studies have not yet managed to establish its pathogenesis, since it is highly variable. Similarly, much remains to be elucidated about plant defense mechanisms against the pathogen. Further knowledge of these two aspects is fundamental for the development of tools and strategies to protect the crop. This review summarizes up-to-date information on the disease’s pathogenesis, symptomatology, and geographical distribution, as well as on the environmental factors that favor infection, host defense mechanisms, and resistant chickpea genotypes. It also outlines existing practices for integrated blight management.

## 1. Introduction

Plant food sources satisfy the dietary needs of around 80% of the global population, which currently stands at roughly 7500 million people. This figure is expected to climb to 9000 million by 2050 [1]. To meet the associated caloric and protein demands, agricultural production will have to reach unprecedented heights. This will only be possible through strategies that boost productivity while ensuring sustainability [2]. Such strategies include soil and water conservation, improvements in drainage, efficient nutrient management, crop diversification (with the inclusion of restorative crops), crop rotation (by alternating legumes with crops that do not fix nitrogen), adequate fertilization that reduces the use of artificial chemicals and incorporates rhizobacterial products, biological pest control, and integrated disease management [3,4].

Legumes cover approximately 10% of the global cropping area. Chickpea (*Cicer arietinum* L.) is one of the most cultivated legumes worldwide, after the common bean (*Phaseolus vulgaris* L.) and pea (*Pisum sativum* L.) [5,6]. It grows in subtropical, temperate, arid, and semiarid regions in at least 50 countries [7,8]. Almost 15% of the global legume production and of the cropland covered by legumes correspond to chickpea (17.2 million tonnes annually and 17.8 million hectares, respectively) [9]. The seed’s nutritional composition is well-balanced for human consumption: it consists of 19.3% protein, 43.3% carbohydrates, and 6% lipids. It is also rich in vitamins (B9, A, B2, and B6) and minerals (iron and zinc), and is thus an ideal dietary complement for cereal grains [10,11].

The main disadvantage of chickpea lies in the variability of its yields, which are affected by abiotic and biotic factors such as drought and fungal diseases [12]. Among the latter, one of the most widespread is Ascochyta blight. Caused by *Ascochyta rabiei*, it can be detected in leaves, roots, and other plant tissues [13], and can lead to total yield losses or significantly reduce quality in vulnerable, untreated cultivars [14,15]. In Australia, for instance, it is responsible for losses averaging AUD 4.8 million a year [16].

The present review summarizes up-to-date information on Ascochyta blight disease. The topic is approached from several perspectives. Relevant aspects of fungus and plant biology are covered (such as fungal reproduction and plant defense mechanisms), as well as strategies for integrated disease management. Among the latter, we have included those related to biological control, which have been less explored but show great promise. 

We believe this review could serve as a useful data compilation for researchers with an interest in the topic, and that it could encourage further research on Ascochyta blight at a genomic, physiological, and agricultural level.

## 2. Host Plants for *Ascochyta rabiei*

Chickpea is the most frequent host of *A. rabiei*, and therefore the most susceptible crop to Ascochyta blight. Lentil (*Lens culinaris*), cowpea (*Vigna unguiculata*), pea (*Pisum sativum*), and the common bean (*Phaseolus vulgaris*) have also been infected with *A. rabiei* under laboratory conditions, which means that the fungus has a certain degree of pathogenicity in these species [17,18,19,20]. Other hosts include *Medicago sativa*, *Melilotus albus*, *Lactuca serriola*, and *Thlaspi arvense* [21], all of which are cultivated in chickpea-growing areas. Less commonly, *A. rabiei* has been isolated from *Brassica nigra*, *Lamium amplexicaule*, *Descurainia sophia*, *Galium aparine*, and *Triticum aestivum*. These crops are grown on fields where chickpea residues from previous seasons may remain on the soil surface [22]. In general, however, the disease is rare in hosts other than chickpea. When this does occur, it is dormant (i.e., asymptomatic) or mild, but the plants may act as pathogen reservoirs or “green bridges” [23].

A better understanding of *A. rabiei*’s host range is essential to manage Ascochyta blight and effectively disrupt its cycle, particularly in regions where chickpea is a staple crop, the disease is endemic, or ascospores are one of the primary sources of initial infection. Ascochyta blight management, therefore, depends partly on appropriately identifying the diversity of host plants, as well as the variations in pathogenicity from one plant species to another [24].

## 3. Geographical Distribution and Time of Emergence of Ascochyta Blight

Archeological findings place the beginnings of chickpea cultivation as far back as 7500–6800 BCE, in the Middle East. Chickpea is thus one of the earliest domesticated crops, and today grows in over 50 countries in Asia, Africa, Europe, Oceania, and North and South America [9]. As mentioned above, *A. rabiei* is responsible for one of the most devastating fungal diseases that affect this crop [22]. Ascochyta blight was first identified in 1911, in what was then India’s North-West Frontier Province (currently Pakistan) [25]. Since then, it has spread to most chickpea-growing areas around the world, including 40 countries in western Asia, southern Europe, northern Africa, certain countries in the Americas, and parts of Oceania [25]. No cases have been reported in Nepal, Myanmar, Bolivia, Peru, Chile, Colombia, Libya, Malawi, Zambia, Sudan, Uganda, or the Balkans [26].

In Pakistan and the Indian subcontinent, the first signs of disease usually appear in the winter (February and March). In the north of India in particular, Ascochyta blight is predominant in densely cultivated areas. On the other hand, chickpea is sown towards the end of winter in the Mediterranean, western Asia, and northern Africa. This is why initial symptoms in these regions tend to appear in the spring, when the weather is warm and humid (between March and May), and they may still be visible at the end of the crop cycle (November–December). 

In South America, Argentina is one of the countries where chickpea has been gaining the most traction. Ascochyta blight was first reported here during the 2011/2012 season [23], when chickpea cultivation reached its peak in terms of land sown (120,000 hectares). That year, 7% of the international chickpea market was produced in Argentina, and national exports exceeded those by two leading exporters, the US and Canada [27]. *A. rabiei* was introduced into the country through seeds, the only long-distance dissemination route. The central provinces of Córdoba and Buenos Aires suffered the most, and symptoms consisted frequently of withering, blight, and plant death. Crop deaths were initially recorded in November 2011, a time when pods commonly begin to develop [23]. Over time, the disease has become more severe in central-north Córdoba and has even caused the loss of complete plots, which is why it is now considered a major limiting factor for chickpea cultivation in Argentina [28].

## 4. Characteristics of Ascochyta Blight in Chickpea

### 4.1. Causal Agent

**Figure 1:**
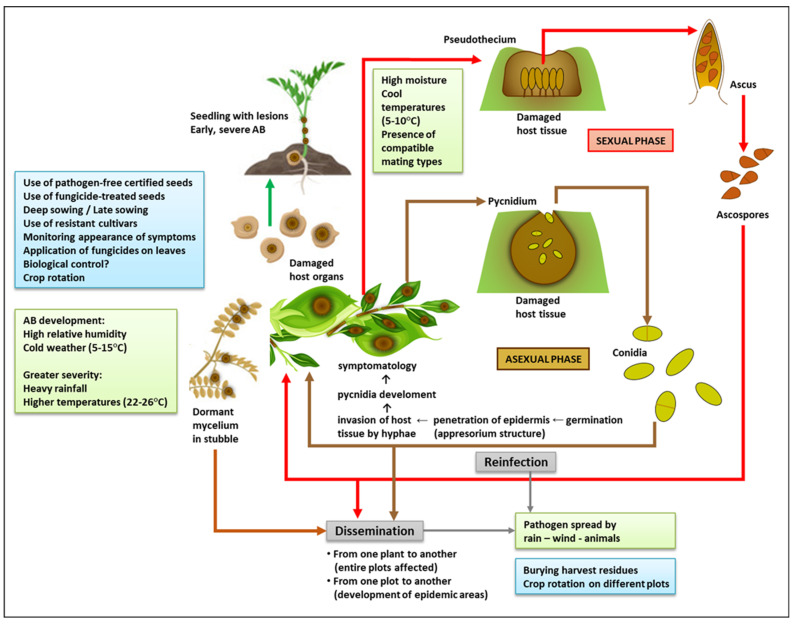
Integrated view of Ascochyta blight (AB) disease: life cycle, predisposing environmental conditions (green boxes), and management practices (light blue boxes). To make this figure, some images were downloaded from https://www.vectorstock.com/royalty-free-vector/green-pod-chickpea-as-annual-legume-plant-vector-31018799 and https://www.vectorstock.com/royalty-free-vector/chickpea-plant-growth-stages-infographic-elements-vector-25494313 (accessed on 5 January 2023).

*A. rabiei,* the causal agent of Ascochyta blight, can attack all parts of the plant and lead to necrosis, tissue collapse, and therefore to the death of organs or even the whole plant. This may translate into total or major yield losses [15]. *A. rabiei* (Pass.) Labr. is the name of the anamorph of the fungus during its imperfect or asexual stage. Alternative names for the anamorph are *Phyllosticta rabiei* (Pass.) or *Phoma rabiei* (Pass.). It also has a sexual reproductive stage or teleomorph, known as *Didymella rabiei* (Kovachevski) v. Arx (also *Mycosphaerella rabiei* Kovachevski) (Figure 1). The key taxonomic descriptors for this fungus are phylum Ascomycota, class Dothideomycetes, order Pleosporales, family Didymellaceae [25,29,30].

When the weather is cool and wet, the pseudothecium (the sexual fruiting body) is formed within infected plant tissues. It is a dark brown/black globular structure, akin to a loculated perithecium, which measures approximately 120–270 μm. It contains several pedicellate, curved, cylindrical-clavate asci or sac-like structures (about 48–70 × 9–13.7 μm in size). Within each ascus, there are eight small septate ascospores (12.5–19.0 × 6.7–7.6 μm), each made up of two cells of different size. The bigger cell is prominently formed on the septum itself [31,32]. Since pseudothecia remain inside the tissue after it has died (i.e., inside stubble), ascospores are easily spread to other plots by the wind during the spring and summer [31,32]. The teleomorph is not found in areas where warm conditions prevail after the summer and into the chickpea growing season.

### 4.2. Environmental Conditions That Favor the Fungus

The prevalence and spread of the disease are critically influenced by factors such as relative humidity, temperature, and wind [33,34]. Pycnidia, the asexual fruiting bodies of the fungus, can survive more than two years in crop residues if the temperature allows it (10–35 °C with high relative humidity), and the disease typically develops in humid, cold weather (5–15 °C) [35,36]. Temperatures ranging from 22 °C to 26 °C accompanied by heavy rainfall can also favor the appearance of the disease at all stages of the crop cycle (from seedling to pod) [37] (Figure 1). The pathogen is transmitted through stubble and seeds that become infected when it rains in windy weather, through leaves, and by insects and other animals. During the crop cycle, the wind transfers it from infected to uninfected plants, which leads to the formation of spotted areas that may progressively cover entire plots [14]. This progression may be limited in dry weather, but occurs rapidly in humid conditions. Fruiting bodies grow fast at 20 °C [33]. Long periods of cold and humidity are the most propitious for oozing conidia that are spread from pycnidia by the rain [31]. The persistence of such environmental conditions and the presence of compatible mating types [25] favor the sexual cycle, which makes it more likely for the disease to turn into an epidemic. The dissemination distance of the different spores is crucial to determine the spread of the disease. Asexual spores (conidia) disseminate across short distances, and they depend mainly on windy weather and rain splash to spot or infect plots. On the other hand, sexual spores (ascospores) spread through the air, which means they can probably travel greater distances and create epidemic areas of Ascochyta blight disease [38]. These differences are worth considering as part of management and control strategies.

### 4.3. Pathogenesis and Symptomatology

*A. rabiei* is a necrotrophic fungus, and its mycelia can remain dormant in stubble for up to 3–4 years. The chickpea seed is crucial for the continuity of its biological cycle from one generation to the next, since the fungus survives for over five months on the episperm, the cotyledons, and the embryo. Infected grains are therefore the safest and most efficient means for the pathogen to spread and persist [39].

Chickpea is usually affected by *A. rabiei* during flowering and pod formation [40]. The active fungus can directly target all plant tissues (leaf, petiole, stem, pods, and seeds). Pathogenesis relies on conidia being deposited on the plant surface, their subsequent germination, and the formation of an appressorium-like structure at the tip of the germinal tube. This structure, which penetrates the epidermis, makes it possible for hyphae to invade the adjacent subepidermal tissue and for pycnidia to develop [41,42]. Pycnidia are produced inside the leaves, stem petioles and pods, and even the seeds. They are dark brown globose structures, 140–200 mm in diameter, and have a prominent ostiole [14]. The process may be more or less virulent depending on the aggressiveness of the fungal strain and the tolerance of the infected cultivar [43,44].

The initial symptoms are small necrotic spots. On leaves and pods, necrotic spots are observed as black concentric circles that form round or oval lesions (between 2 and 14 mm in size). On stems, these spots are more oblong (2–30 mm) [26]. Necrosis occurs when the host tissue is degraded by three potent phytotoxins (solanapyrone A, B, and C). Certain enzymes also degrade the plant cell wall (cutinase, pectinase, polygalacturonase, xylanase), while others favor host colonization by acquiring nutrients through the digestion of plant matter (peptidases, lipases), and by inactivating host defenses (dehydrogenases, peroxidases, oxidoreductases) [45]. The production of these virulence factors is related to *A. rabiei*’s necrotrophic lifestyle, and the levels produced depend directly on the strain’s aggressiveness [45,46]. When infection in the leaves is severe, the entire plant dries up and collapses. High temperatures prevent the disease from progressing to that point, but the tissues remain infected and discrete lesions are still observable [47].

If a pod is infected when it has only just started forming, it becomes unviable for seeds to grow within it [48,49,50]. Once grain formation has begun, the pathogen can easily penetrate the pod wall and settle inside the seed, which significantly enhances its chances of survival and dissemination [51]. Whether superficially or internally infected, seeds may either show no symptoms (if the infection is mild) or shrivel and display dark lesions of different shapes and sizes [52]. As in the case of other plant parts, these lesions are created by brown/black pycnidia growing concentrically (95–220 μm). When hydrated, these pycnidia create a viscous mass that releases conidia [23]. Sowing infected seeds can lead to the establishment of disease at an early stage of plant growth, if the weather conditions are favorable for conidial germination. The emerging seedlings will have dark brown lesions at the base of the stem [53]. Spots formed on the stem at this time are the most serious, since they prevent sap from circulating and thus lead to early death [9] (Figure 1).

### 4.4. Plant Defense Mechanisms

Finding chickpea varieties that may be resistant to Ascochyta blight is complicated by a series of factors: the low resistance coded by the primary gene set; the complex genetic basis for resistance conferred by several quantitative trait loci (QTL); the variability of pathogen populations; and the emergence of new pathotypes due to natural recombinations that take place during *A. rabiei*’s cycle of sexual reproduction [13,38]. Nevertheless, chickpea is known to have an intricate defense response against the fungus. This response varies depending on the crop’s resistance/susceptibility genotype, the fungal strain infecting it, and the environmental factors at the time of infection. Most studies on this topic have focused on the differences in gene expression from one cultivar to another upon exposure to the pathogen, which influences pathogenesis after initial infection [54,55].

Enzymes are among the protective factors that have been identified so far. These include several pathogenesis-related (PR) proteins, such as one acting as a β-1,3-glucanase [56,57], and chitinase, which mediates the degradation of the fungal cell wall [58]. Others, such as polyphenyloxidase, catalase [59], and copper amine oxidase (CuAO), are produced in the epidermis and xylem vessels [60] and are involved in the oxidative burst. This is a process through which the superoxide dismutase copper chaperone precursor (SDCC) and glutathione S-transferase (GST) are downregulated to increase H_2_O_2_ levels [53]. Both the oxidative burst and the accumulation of reactive oxygen species (ROS) make up a complex defense mechanism which is related to the hypersensitive response (HR), a way for the plant to prevent microbial propagation through programmed cell death. In resistant cultivars, HR has been detected in association with the synthesis of metabolic enzymes, such as those involved in the production of phytoalexin, an antimicrobial compound [43]. However, certain *A. rabiei* pathovars have been observed to degrade phytoalexins synthesized by chickpea [61]. Other proteins are overexpressed in plants as part of the defense response against *A. rabiei*, such as an environmental stress-inducible protein (ESP), a Ca-binding protein, and several others which remain unidentified [53].

Defensive barriers play their part in plant defense as well. Cell walls, for instance, tend to become more stable when exposed to stressful conditions. Infection with *A. rabiei* induces the expression of the gene that encodes snakin-2 (SN2), a cysteine-rich peptide with a broad antimicrobial spectrum [53], and the production of proline-rich proteins (PRPs) that strengthen the structure of the primary cell wall [62]. This structure is reinforced further by ROS synthesis and the creation of disulphide bridges [63].

Host plants also defend themselves against *A. rabiei* through the complex upregulation of genes linked to transcription factors. These genes encode products such as (i) a disease-resistance response protein, DRG49-C [53]; (ii) a leucine-zipper protein (LZP), probably involved in the synthesis of PR proteins and the production of salicylic acid [53,64]; (iii) the polymorphic antigen membrane protein PAMP, associated with the transcription of defense genes related to the metabolism of polyamines and nicotianamines [54]; (iv) the ethylene receptor gene ERG, named CaETR1 in *C. arietinum* L. and located in QTLAR1 [65]; (v) a pathogenesis-related transcription factor, TF1082, linked to the response of ethylene during infection [66]; (vi) the CARNAC transcription factor associated with plant development and defense [67]; and (vii) a resistance gene analog, RGA4, which belongs to chickpea RGA families and has nucleotide binding sites and leucine-rich repeat domains (NBS-LRR) [13,68]. 

Overall, and in spite of the severity of Ascochyta blight, chickpea can deploy several defense strategies against it, probably in a coordinated manner. The complexity of the response depends on multiple biotic and abiotic factors, and a better understanding of this complexity relies on acquiring further knowledge about the genes involved. The ultimate aim, and perhaps the most difficult to achieve, is to harness these mechanisms for the design of chickpea varieties that will be resistant to blight pathovars under different environmental conditions.

## 5. Disease Management

To effectively manage crop disease, the plant population must be continuously monitored for the appearance and progression of pathogens. This is crucial for eradicating them or reducing their inocula. In the case of Ascochyta blight in chickpea, the objective is to prevent infection from affecting entire plots. Using the host plant’s own immune response to do this could be eco-friendly and economical. However, the task has been complicated by the emergence of new pathovars which are resistant to this response. Upon the arrival of the disease in Argentina, researchers sought to elucidate how different cultivars behaved when exposed to locally isolated variants of the pathogen. Today, we know that the cultivars grown here are vulnerable or moderately vulnerable to the disease [23], and that the best way to minimize its impact and ensure sustainable yields is through integrated management practices. These can include burying harvest residues, eliminating the inocula transmitted through seeds, and planting resistant varieties. The International Center for Agricultural Research in the Dry Areas (ICARDA) [69] and the International Crops Research Institute for the Semi-Arid Tropics (ICRISAT) [70] have released many Ascochyta blight-resistant cultivars [26]. These two organizations not only promote planting these cultivars, but also the use of disease-free seeds, the application of fungicides on seeds and leaves, the rotation of crops every three years, and stubble control. 

### 5.1. Types of Disease Control

Successful management of Ascochyta blight is made difficult by the lack of cultivars with maximum resistance and of highly effective fungicides. The problem is compounded by weather conditions that are extremely favorable to the development of the disease [71]. In general, control strategies can be grouped into three categories: cultural, chemical (selective fungicides), and biological (biocontrol agents) [13].

#### 5.1.1. Cultural Control

Cultural practices, which are aimed at reducing inocula by making the crop ecosystem less hospitable for pathogens, are the first line of defense against Ascochyta blight and other crop diseases. Most important of all is sowing healthy seeds, i.e., seeds which have been certified as pathogen-free. Crop rotation may also be implemented, by alternating chickpea with other crops that do not host the fungus (such as cereals) at least every three years. Deep sowing can further protect the plant, and late sowing can decrease plant growth and thus the incidence of the disease. Increasing the space between furrows and planting cultivars that grow compactly and erectly can create more unfavorable conditions for the fungus to thrive (less water condensation or humidity) [38,72]. Potassium fertilizers may enhance chickpea’s robustness against infection, particularly in soils with a high nitrogen content [73]. Burying chickpea stubble can inhibit the formation and maturation of teleomorphs [74]. Stubble may be burned for the same purpose, but this can deplete the soil of organic matter and essential nutrients. Used in combination and on a schedule, all these practices can contribute to limiting the impact of Ascochyta blight on chickpea [75].

#### 5.1.2. Resistance in the Host Plant

Using chickpea’s own genetic resources against Ascochyta blight is one of the most sustainable and economical ways to minimize yield losses due to the disease [76]. These resources, known collectively as host plant resistance (HPR), can be taken advantage of on their own or as the main component in integrated disease management programs. To do so, resistant cultivars must be identified through reliable and reproducible techniques. Varying results have been obtained with such techniques on the field and in the greenhouse, depending on factors such as inoculum concentration, inoculation method, plant age at the time of inoculation, and environmental conditions such as temperature, humidity, and photoperiod [77,78,79,80]. Changes in any of these elements can affect a technique’s efficacy, which is why the only way to ensure its reliability and reproducibility is to identify and standardize the variables that influence infection. 

Some of the techniques which have been tested to find resistant chickpea cultivars include field screening under natural conditions [22]; assessment of genotypes under temperature and relative humidity controlled through fogger irrigation [81]; a “mini-dome” assay [82] that successfully detected Ascochyta blight-resistant germplasm [22,83]; and the identification of genes in different *Cicer* species that may serve as novel sources of resistance [16].

A widely validated method of detection used by researchers is the one adopted by ICARDA’s program for crop improvement. It consists of growing resistant germplasm under stressful conditions with exposure to the disease. More specifically, plants in a nursery are inoculated with diseased chickpea residues and artificial spore suspensions, and then two classification methods can be applied [13,82,84]. The first consists of calculating the percentage of infected specimens within a given genotype. The genotype is then assigned a number on a scale from 1 to 9 that indicates its vulnerability to infection [85]. Genotypes assigned scores between 1 and 3 (0–10% infection cases) are considered resistant to *A. rabiei*, and the rest of the lines are eliminated. A similar scale was designed in parallel by Manjunatha and Saifulla (2013) [86], also with the aim of classifying chickpea genotypes as resistant, moderately resistant, tolerant, and susceptible to Ascochyta blight. The second classification method, which is more finely targeted, determines the vulnerability of genotypes depending on the percentage of infected leaves in all specimens [87].

In 2001, the moderately resistant Howzat chickpea variety was released in Australia. Breeders then obtained the desi and kabuli types, which are more resistant than those designed by ICRISAT and ICARDA and than all existing Australian ones. Countries such as India, Pakistan, Syria, the US, and Canada have also released Ascochyta blight-resistant cultivars [88]. A thorough list of *A. rabiei*-resistant chickpea germplasms has been found in Islam et al. (2017) [83]. In more recent years, other resistant germplasms have been described [84,89,90,91,92,93]. The search for resistant varieties is still ongoing, with genomic studies currently focusing on identifying and characterizing morphological, biochemical, and molecular traits (molecular breeding) [94,95].

#### 5.1.3. Chemical Control

Genetic resistance, healthy seed sowing, and intelligent cultural practices are not enough to manage Ascochyta blight, and should be combined with chemical control methods such as seed treatment and the application of foliar fungicides [96]. Three types of systemic fungicides are mainly used: demethylation inhibitors (DMIs, triazoles); succinate dehydrogenase inhibitors (SDHIs, boscalid); and quinone outside inhibitors (QoI, pyraclostrobin or azoxystrobin). Specific formulations of metalaxyl, captan, tiabendazole, benomyl, fluxapyroxad, and pyraclostrobin are also applied on chickpea seeds, as well as mixed formulations [96,97].

Protective fungicides, such as those containing chlorothalonil, are effective before flowering and before the furrow is covered by crop growth [96]. The time of application of all fungicides is critical once symptoms have appeared [98]. When the risk of disease is high (i.e., when the environmental conditions are favorable, or when there have been reports of infection in neighboring areas), aggressive application might make sense from an economic standpoint. Nevertheless, care should be taken to prevent the emergence of fungicide-resistant pathogenic isolates. For instance, several cases have been reported of *A. rabiei* resisting strobilurin-based products. These products (and others with mixed modes of action) must be combined with other active ingredients for effective control [96]. DMIs, SDHIs, and multi-target fungicides are still efficient in those cases where strobilurin is not. DMIs based on prothioconazole are usually successful under intense disease pressure, and so is boscalid under moderate disease pressure. In general, rotating fungicides with different modes of action is critical to prevent the fungus from developing resistance.

The polycyclic nature of Ascochyta blight and the fact that its severity can increase quickly under the right environmental conditions mean that repeated fungicide applications may be necessary [96,99]. These repeated applications make fungicides a more costly method of control than others [100]. Moreover, fungicides can be toxic for humans and wildlife, and they can contaminate food and ecosystems [101]. There is therefore an urgent need for alternative bioproducts that may contribute to the management of blight and other phytopathogenic mycoses [102]. As the situation stands today, an adequate use of the available fungicidal agents is paramount to stop fungal propagation, which can be rapid due to the existence of spores in the air, the speed of reproduction, and the fact that many fungal species can reproduce sexually. 

#### 5.1.4. Biological Control

As mentioned in the previous section, alternative disease control strategies are necessary to bypass the toxicity of traditional chemical methods while ensuring long-term effectiveness and sustainable yields. One such alternative is the use of biological control agents, i.e., microorganisms that can naturally antagonize plant pathogens [103]. These microorganisms (mainly fungi and bacteria) are typically isolated from the soil or the plant, and their biocontrol abilities against a given pathogen are then tested in vitro. Many research lines currently focus on plant growth-promoting rhizobacteria (PGPR) [4,104], which have been reported to protect plants against harmful bacteria, fungi, virus, insects, and nematodes [105]. 

Effective biocontrol agents are usually able to fight a pathogen through several mechanisms deployed coordinately. They may quickly colonize the habitat and outcompete the pathogen for space and nutrients; they may parasitize the pathogen [105]; produce enzymes that harm or destroy it (i.e., lytic enzymes) [106]; synthesize water-soluble or volatile antibiotics [107,108]; and/or induce the host’s own systemic resistance [109]. Many members of the bacterial genus *Burkholderia* synthesize antifungal substances that depend on quorum sensing [110]. Similarly, some *Pseudomonas* species produce secondary metabolites (such as pigments and heterocyclic compounds, such as phenazine) which have broad-spectrum antifungal properties. Certain fungal species have also shown promising activity against Ascochyta blight in vitro [111] (Table 1).

Plants themselves produce secondary metabolites that can have antifungal and antimicrobial activity [111,112,113]. For this reason, plant extracts and essential oils are being studied as a natural solution to phytopathogenic disease. Although few of them have already been authorized for their extensive use in agriculture, their main advantage lies in the fact that they are safe and eco-friendly [112,114]. Some of the extracts and oils that have been found to be effective against plant disease are *Aloe vera*, *Magnolia grandiflora*, *Tagetes erecta*, *Thymus vulgaris*, and *Origanum vulgare*.

**Table 1:** Biological agents linked to *A. rabiei* inhibition (summary).

Type of Agent	Name	Activity against *A. rabiei*	Reference
Fungus	*Chaetomium globosum* Cg2	Inhibited the mycelium profusely	[115]
	*Trichoderma viride* TV-5-2	Inhibited growth and sporulation	[115]
	*Acremonium implicatum (Isolate #1)*	Inhibited and lysed the mycelum	[115]
	*Acremonium implicatum (Isolate* #*2)*	Inhibited the mycelium	[115]
	*Trichoderma harzianum*	Inhibited the mycelium and stopped growth after 7 days of exposure	[116]
	*T. harzianum* T15	Produces chitinase and β-1,3-glucanase	[117]
	*Aureobasidium pullulans*	Reduced lesions when applied on plants	[118]
Bacterium	*Pseudomonas fluorescens*	Inhibited the mycelium (>30%)	[119]
	*Pseudomona putida*	Inhibited the mycelium (>30%)	[119]
	*Bulkholderia multivorans*	Inhibited the mycelium (>30%)	[119]
	*Mesorhizobium ciceri*	Inhibited the mycelium (>30%)	[119]
	*Burkholderia ambifaria*	Inhibited the mycelium (>30%)	[120]
	*Burkholderia ambifaria*	Inhibited disease development in plants inoculated in vitro	[120]
	*Bacillus megaterium*	Inhibited growth and germination of spores	[121]
Plant extract	*Chenopodium album*	Inhibited fungal biomass (70%)	[122]

Although the potential of biocontrol agents for the future of agriculture cannot be understated, a factor which may limit their success is their sensitivity to elements that are hard to control (temperature, humidity, acidity, UV light, etc.). In other words, their efficacy can be wildly variable depending on the existing conditions. In the specific case of Ascochyta blight, biological activity against it has been seldom studied and only in vitro (Table 1). Reviews such as the present one might hopefully draw attention to the critical need for further research to find appropriate biocontrol strategies against this widespread disease.

### 5.2. Integrated Disease Management

The best strategy against Ascochyta blight nowadays is to integrate all the resources available, i.e., to combine the sowing of moderately resistant cultivars with smart field management strategies and minimal applications of foliar fungicides (Figure 1). The same scheme is adaptable to deal with other fungi that affect chickpea and other crops. To summarize, integrated management practices comprise:

1. Planting pathogen-free seeds, which decreases transmission to seedlings.
2. Treating seeds with fungicides.
3. Rotating crops every three years (at least) to reduce inoculum in the stubble, while making sure to eliminate volunteer plants and/or weeds that may act as disease reservoirs. Alternating the cultivation of chickpea with that of cereals such as barley, which do not host the pathogen, has been shown to be effective at limiting fungal propagation. In warm and humid areas where stubble decomposes fast, only 1–2 years of alternative crops in between chickpea harvests are enough to bring the inoculum down to manageable levels.
4. Planting resistant genotypes whenever possible.
5. Applying fungicides wisely. In general, the first application should be performed between 4 and 6 weeks after sowing. Moderately resistant cultivars require spraying 2 to 4 times throughout their growth. Moderately susceptible cultivars need spraying every 2–3 weeks. In the case of vulnerable cultivars, the frequency of application should be roughly once every two weeks. Most fungicides with known efficacy act better preventively, so they must be applied before infections appear in those areas where growing seasons are short.
6. Deep plowing fields where chickpea has grown before, to bury infected stubble.
7. Disinfecting machinery, vehicles, tools, and footwear, as appropriate.
8. Making crops stronger and less susceptible to disease through adequate nutrition. The soil must be monitored for nutritional deficiencies and may be supplemented with additional nutrients. The incidence of the disease may be reduced by adding potassium and phosphorus to soils with a high nitrogen content, and by applying micronutrients to leaves during the reproductive stages. A combination of 40–60 kg of potassium, 20 kg of nitrogen, and 40 kg of phosphorus has been reported to increase yields and minimize disease severity [123].
9. Isolating previously sown plots and sowing away from where chickpea plants have grown before, to reduce the density of ascospores released from infected residues in areas where they are the main source of inoculum.

## 6. Conclusions and Perspectives

The severity of blight caused by *A. rabiei* in chickpea is a consequence of its high aggressiveness and long-term persistence in the environment, and can only be tackled through integrated management practices.

Interdisciplinary work is required to design predictive models based on the complex interactions between the disease and environmental variables, so that better management decisions can be made. These decisions would also be served by further research into the genetics, ecology, host-pathogen interactions, and plant defense mechanisms that play a part in the appearance and progression of blight. Such research would also benefit the development of novel resistant cultivars and of biological tools to reduce the phytopathogen’s harmful impact.
